# Genetic Polymorphisms in Rheumatoid Arthritis: From GWAS-Identified Loci to Functional, Biomarker, and Pharmacogenomic Insights

**DOI:** 10.3390/genes17101175

**Published:** 2026-09-24

**Authors:** Paulina Plewa, Hanna Ostałowska, Zuzanna Leciej, Jacek Szulc, Aleksander Cierlecki, Radosław Birger, Andrzej Pawlik

**Affiliations:** 1 Department of Physiology, Pomeranian Medical University, 70-111 Szczecin, Poland; paulina.plewa@op.pl (P.P.); hannaostalowska@gmail.com (H.O.); leciejzuzanna@gmail.com (Z.L.); 90890@student.pum.edu.pl (A.C.); 2 Department of Experimental and Clinical Pharmacology, Pomeranian Medical University, 70-111 Szczecin, Poland; radoslaw.birger@pum.edu.pl; 3 Laboratory of Pharmacodynamics, Pomeranian Medical University, 71-244 Szczecin, Poland

**Keywords:** rheumatoid arthritis, genetic polymorphisms, genome-wide association studies (GWAS), HLA-DRB1, single nucleotide polymorphisms (SNPs)

## Abstract

Rheumatoid arthritis (RA) is a chronic autoimmune disease with a complex, multifactorial aetiology, in which both environmental factors and genetic predisposition play a significant role. This narrative review summarises the genetic polymorphisms associated with RA that have been identified by genome-wide association studies (GWAS). Its aim is to synthesise the loci and single-nucleotide polymorphisms (SNPs) reported to date, to relate them to the immunological mechanisms through which they are thought to act, and to assess their emerging diagnostic, prognostic, and pharmacogenomic applications. Its scope covers large-scale GWAS and multi-ancestry meta-analyses together with the candidate-gene, functional, and pharmacogenomic studies required for their interpretation. Three principal conclusions emerge from the literature reviewed. First, the genetic architecture of RA remains dominated by the HLA-DRB1 shared epitope, which is associated primarily with seropositive, anti-citrullinated protein antibody-positive disease, whereas the numerous non-HLA loci identified to date each confer only a modest individual effect. Second, these non-HLA variants converge on a limited number of biological processes—the activation thresholds of T and B lymphocytes, NF-κB and JAK–STAT cytokine signalling, and the invasive behaviour of fibroblast-like synoviocytes—so that their value lies less in the prediction of risk by individual variants than in identifying pathways amenable to therapeutic targeting. Third, the most readily translatable findings are pharmacogenomic. Variants affecting adenosine signalling and nucleotide metabolism modulate the response to methotrexate. Several polymorphisms predict the response to TNFα inhibitors. Polygenic risk scores are beginning to support the differential diagnosis of early inflammatory arthritis. Progress remains constrained by the over-representation of European-ancestry cohorts, by the difficulty of moving from an associated locus to a causal variant and its target gene, and by the limited replication of pharmacogenomic associations. Integration of genomic with transcriptomic, epigenomic, proteomic, and metabolomic data, supported by machine learning approaches, is therefore required before genotype-guided management of RA can enter routine clinical practice.

## 1. Introduction

Rheumatoid arthritis (RA) is an autoimmune inflammatory disease that can affect the entire organism, with the joints as the first target. Apart from the joints, RA can also manifest in other organs and systems like the skin, eyes, kidneys, cardiovascular system, respiratory system, gastrointestinal system, and nervous system [1]. According to current estimates, approximately 0.25% of the global population is affected by RA. The prevalence of RA is up to 1% in Northern Europe and the United States, and in some isolated populations it can be even higher. Women have a two to three times higher general risk of developing RA than men [2,3]. The course of RA includes activation of the adaptive immune system and the innate immune system. The humoral and cellular response involves the production of autoantibodies, mostly rheumatoid factors (RF) and anti-modified protein antibodies (AMPAs) accompanied by migration of T and B cells into the synovium. Furthermore, there is a strong tissue activation of the monocyte/macrophage system, indicative of the innate immune system response. Together, these factors lead to the development of inflammation inside the joints [4].

Genetics contributes significantly to RA development and is strongly connected to many environmental triggers such as cigarette smoking (the main risk factor), diet, infectious agents, and obesity. Genetic factors account for about 60% of the variation in a person’s susceptibility to RA. The occurrence of RA in a first-degree relative can increase the risk of disease development by up to two to five times, indicating escalated prevalence within families [5,6]. Certain alleles of the HLA-DRB1 gene, located within the human leucocyte antigen (HLA) region on chromosome 6, are strongly associated with susceptibility to RA. HLA genes encode components of major histocompatibility complex (MHC) class II molecules, which play an important role in immune responses. In addition to HLA-DRB1, numerous genetic variants, including single-nucleotide polymorphisms (SNPs) located outside the HLA region (non-HLA loci), have also been associated with RA susceptibility. These variants may influence immune pathways and contribute to an individual’s genetic risk of developing RA [7].

Multiple genetic variants, including SNPs, are associated with susceptibility to common diseases such as RA. These disease-associated variants can be identified through genome-wide association studies (GWAS), which typically involve analysing genetic variants across the genome to identify statistical associations between genetic variation and disease phenotypes. In contrast to the pre-GWAS era, when candidate genes were selected based on existing biological knowledge and analyses were generally limited to variants located within or near those genes, GWAS provide a hypothesis-free approach to identifying genetic associations across the genome. Candidate-gene studies were also often limited by a lack of reproducibility. In contrast, GWAS have identified thousands of robust and reproducible genetic associations with diseases and other complex traits. Depending on the study, hundreds of thousands to millions of genetic variants are analysed in large numbers of individuals to identify genotype–phenotype associations. GWAS findings can contribute to the identification of genetic factors associated with disease susceptibility and may inform the development of approaches to assessing genetic risk [8,9].

Several comprehensive reviews addressing the genetics of rheumatoid arthritis—covering susceptibility loci, disease severity, treatment response, functional mechanisms, and pharmacogenomics—have been published very recently [5,10,11]. The present review differs from these publications in two respects. First, rather than focusing on a single axis (e.g., susceptibility alone, or pharmacogenomics alone), it synthesises GWAS-identified risk loci together with the downstream candidate-gene, functional, and pharmacogenetic evidence generated for those same loci, tracing a continuous line from discovery to mechanism to clinical application within one narrative. Second, it places particular emphasis on multiomic approaches into polygenic risk prediction and biomarker discovery, an area that has expanded substantially. Because much of the evidence discussed originates from candidate-gene, case-control, functional, and pharmacogenetic studies performed as follow-up to GWAS discovery rather than from GWAS analyses themselves, we use the term “GWAS-identified”.

This review explores the SNPs associated with RA identified through GWAS and discusses their biological and clinical impact on disease development.

## 2. Materials and Methods

This review is based on a non-systematic literature search of the Pub-Med/MEDLINE, Scopus, and Google Scholar databases, restricted to English-language publications from 2002 to 2026. Search terms combined “rheumatoid arthritis” with “genome-wide association study”, “GWAS”, “polymorphism”, “SNP”, “single nucleotide polymorphism”, and the names of the individual genes and loci discussed in Section 4 (e.g., PTPN22, STAT4, CTLA4, TNFAIP3, TRAF1, IL6R, IL2RA, CCR6). Priority was given to genome-wide association studies, large case-control replication studies, and mechanistic/functional studies published within the last five years, supplemented by earlier foundational studies where these establish the biological rationale for a locus. Reviews, meta-analyses, and primary research articles were eligible; conference abstracts and non-peer-reviewed pre-prints were excluded unless no peer-reviewed alternative was available.

## 3. Genome-Wide Association Studies in Rheumatoid Arthritis

### 3.1. Principles and Methodology of GWAS

GWAS is a research strategy used to identify genetic variants whose occurrence is statistically associated with a specific phenotypic trait or disease, based on the systematic interrogation of large numbers of variants across the genome, most commonly SNPs [12]. Groups of correlated SNPs showing a significant association with the phenotype define so-called risk loci. The objective of a GWAS is therefore to detect association rather than to demonstrate causality. Identification of the causal variant and of the mechanism through which it acts requires fine-mapping, assessments of linkage disequilibrium (LD), integration with functional data, and experimental validation [12]. Early applications of this strategy—the identification of a myocardial infarction susceptibility region on chromosome 6p21 [13] and of CFH variants in age-related macular degeneration [14]—established the genome-wide approach as a means of dissecting complex diseases.

In practice, a GWAS proceeds through several sequential stages (Figure 1): collection of phenotypic and genotypic data, genotyping by array or sequencing, quality control, phasing and imputation against a reference panel, and association testing of individual variants using regression models that may include covariates such as age, sex, and genetic ancestry. Because hundreds of thousands to millions of variants are tested simultaneously, a genome-wide significance threshold of *p* < 5 × 10^−8^ is conventionally applied in order to limit false-positive findings [12].

Interpretation is complicated by LD. Variants located in close proximity are co-inherited more often than expected by chance, so that a variant which is not itself causal may show a strong association simply because it is correlated with the causal variant. For this reason, GWAS findings are usually interpreted at the level of loci rather than of single SNPs, and defining the causal variant and its target gene requires fine-mapping and functional studies [12].

The principal strength of GWAS is their hypothesis-free, genome-wide scope, which allows loci to be identified without prior assumptions about disease biology and can therefore implicate genes and pathways not previously linked to the phenotype [10,12]. Their results are used to elucidate the biology underlying phenotypes, to estimate heritability and genetic correlations, to predict clinical risk and to prioritise therapeutic targets (Figure 2). Pooling cohorts in GWAS meta-analyses further increases statistical power and the precision of effect-size estimates [12,15].

The limitations of the approach follow from the same design. Associations do not in themselves constitute evidence of causality, and in regions of strong LD the prioritisation of a causal variant among highly correlated SNPs is difficult [12]. The stringent multiple-testing threshold requires very large cohorts, because most variants associated with complex diseases have small individual effects [12,15]. Population structure must be modelled explicitly, since differences in genetic ancestry may correlate with both variant frequency and disease prevalence, and the marked over-representation of European-ancestry cohorts limits the generalizability of findings to other populations [12]. Finally, defining a credible set of variants does not by itself establish the target gene or the functional consequence of a signal, and this biological interpretation remains the principal challenge in the field [12].

### 3.2. Major GWAS Findings in Rheumatoid Arthritis

#### 3.2.1. Number of Identified Risk Loci

The number of risk loci associated with RA has increased alongside the number of study cohorts and the development of multi-ancestry GWAS meta-analyses. Combining data from Korean, Japanese, and European populations comprising 22,628 individuals with RA and 288,644 controls, Ha et al. [16] identified 11 novel risk loci and confirmed 71 previously reported loci outside the MHC region. Overall, the analysis identified 90 distinct association signals shared across East Asian and European populations.

The scale of these analyses has increased in subsequent studies. A multi-ancestry meta-analysis including 276,020 samples from five population groups identified 124 genome-wide significant loci, including 34 previously unreported loci. Genes associated with the newly identified regions included, among others, TNIP2 and TNFRSF11A, which are involved in immune system function, and WISP1, whose function is related to joint tissue biology [17].

The increasing number of identified loci demonstrates that the genetic architecture of RA is complex and involves numerous variants, most of which have relatively small effect sizes. Accordingly, successive GWAS not only expanded the number of known risk loci but also improved the characterisation of similarities and differences between populations and facilitated the identification of potential biological mechanisms involved in RA pathogenesis.

#### 3.2.2. Importance of Large Research Consortia

The increasing number of identified loci is largely attributable to the ability to conduct analyses in very large populations. Individual research centres often lack sufficient sample sizes to detect variants with small effect sizes. Consequently, collaboration among multiple centres and the pooling of data within consortia have become important components of contemporary GWAS research.

A 2022 multi-ancestry meta-analysis provides an example of the use of data from five population groups. Combining the results increased statistical power, enabled the identification of novel loci, and allowed assessments of the potential utility of polygenic risk predictors across different populations [17].

Another example is the study by Saevarsdottir et al. [18], conducted through an international collaboration involving centres in Iceland, Denmark, Sweden, and Norway, including collaboration with deCODE genetics. The analysis included 31,313 individuals with RA and nearly 1 million controls. Integration of GWAS data with multi-omics information, including plasma protein levels and gene expression across different tissues, led to the identification of 37 sequence variants at 34 loci outside the HLA region, including 15 previously unreported variants. Integration of these data also helped narrow the list of potential causal genes associated with individual signals.

Contemporary GWAS consortia therefore serve a role that extends beyond simply increasing sample size. They also facilitate the integration of data from diverse populations and the combination of association results with functional and multi-omics data, thereby enhancing the biological interpretation of identified loci.

#### 3.2.3. Replication of Findings

The replication of findings in independent cohorts is a fundamental component of assessing the reliability of signals identified in GWAS. In particular, confirmation of an observed association in a population independent of the cohort in which the signal was initially detected is of critical importance. For example, Ha et al. [16] identified novel loci and also evaluated previously reported genetic signals. They confirmed 71 previously established loci outside the MHC region in a combined cohort comprising East Asian and European populations.

The study by Mathebula et al. [19] represents an important example of replication in a population that has been underrepresented in genetic studies, namely South-Eastern Bantu-Speaking South Africans. The study comprised 531 individuals with RA and 2653 controls and represented the first RA GWAS conducted in this sub-Saharan African population. The authors confirmed a strong signal in the HLA region involving the rs602457 variant located near HLA-DRB1 as well as two previously reported signals outside the HLA region associated with MMEL1 (rs2843401) and ANKRD55 (rs7731626).

Replication of signals across populations with different genetic ancestries is particularly important for assessing generalisability. At the same time, differences in LD structure between populations may facilitate further narrowing of the regions responsible for observed associations. Expanding GWAS to populations that have historically been underrepresented may therefore improve both the robustness of identified loci and the prospects for their subsequent biological interpretation.

## 4. The Most Important Gene Polymorphisms Associated with RA

### 4.1. HLA Region—The Main Genetic Factor

The Human Leukocyte Antigens (HLA) locus is one of the most important genetic loci when analysing RA susceptibility. The main risk alleles are connected with residues 11, 71, and 74 within HLA-DRB1, which define 16 HLA-DRB1 haplotypes [20]. The predisposing alleles have shared residues in the peptide-binding groove, thus the term “shared epitope” (SE) [21].

However, this data is limited to patients presenting anti-citrullinated protein antibodies (ACPA). The ACPA response is the result of citrullinated peptide presentation to CD4+ T cells, due to citrulline binding by HLA-DRB1.

HLA-DRB1 is responsible for encoding the β chain of HLA-DR, a major histocompatibility complex (MHC) class II molecule [22].

To explicate the subject of ACPA, they are characterised as markers of RA that differ in specificity. They can be divided as non-specific “promiscuous”, with the ability to recognise many antigens, especially citrullinated (via arginine deimination) and homocitrullinated (via lysine carbamylation), or as specific “private” featured by higher specificity and low cross-reactivity [23].

The recently discovered (2026) associations include the following associations: DQA101:02 with multireactive responses towards citrulline-glycine collagen peptides and DRB104:01 to private anti-citrullinated IgG antibodies and reduced collagen-directed multireactivity [23].

The alleles associated with risk are HLA-DRB1*04 (*04:01, *04:04, *04:05, *04:08), HLA-DRB1*10 (*10:01), and HLA-DRB1*01 (*01:01). Allele *14:02 was linked to anti-cyclic citrullinated peptides (anti-CCP) antibody appearance. The protective alleles are recognised as the HLA-DR*13 group (*13:01, *13:02, and *13:04) and HLA-DR*11 group (*11:01, *11:02, and *11:04), followed by non-PR alleles *15:01, *04:07, *03:01, *07:01, and *08:02 [22].

The research has shown that valine at position 11 of HLA-DRB1 in patients with RA is more strongly related to damage observed in imaging examinations (Odds Ratio (OR), 1.75 [95% Confidence Interval (CI), 1.51–2.05], *p* = 4.6 × 10^−13^) and increases overall mortality among patients with inflammatory polyarthritis (HR = 1.16 [95% CI, 1.03–1.31], *p* = 0.01). Moreover, it increases the susceptibility to ACPA-positive RA (OR = 4.26, 95% CI = 3.30–5.49, PGWAS = 7.22 × 10^−29^) in Malays [24].

In the matter of disease-modifying antirheumatic drug (DMARD)-free sustained remission, SE alleles are associated with increased disease persistence, and decreased chance of remission (hazard ratio (HR) = 0.57, 95% confidence interval (95% CI) = 0.42–0.77, *p* = 2.72 × 10^−4^) [25].

There are studies analysing the strongest environmental and genetic risk factors of RA, namely smoking and HLA, and their participation in RA evolution on the basis of pre-arthritis stage. The research has shown that smoking increased the risk of ACPA positivity manifestation in the asymptomatic population (OR 1.37 (95% CI: 1.15 to 1.63)) unlike HLA-SE. Nonetheless, HLA-SE corresponded with inflammatory arthritis (IA) development (HR 1.86 (95% CI: 1.23 to 2.82)), as opposed to smoking. This study portrays that smoking is associated with the early stages of the disease while HLA-SE is linked to clinical progression [26].

### 4.2. Immune Response Regulatory Genes

#### 4.2.1. PTPN22

The Protein Tyrosine Phosphatase Non-Receptor Type 22 (PTPN22) gene is located on the chromosome 1p13.3–13.1 and encodes for Lymphoid-Specific Tyrosine Phosphatase (LYP), which is expressed in T-lymphocytes. Its primary role is to suppress the activity of T cells via negative regulation. Alterations in the structure of LYP, such as SNPs, can imbalance the T-cell function and result in increased susceptibility to autoimmune diseases [27].

Aside from HLA, one of the most significant gene polymorphisms is an arginine to tryptophan substitution R620W (rs2476601) [28].

It is correlated to numerous autoimmune diseases including type 1 diabetes (T1D) and systemic lupus erythematosus (SLE) [29,30].

Additionally, the SNP rs2476601 interferes with the LYP-CSK interaction—a complex of intracellular proteins participating in TCR-mediated signalling suppression, limiting T-cell activation. This alteration diminishes dephosphorylation of the kinases promoting T-cell activation, hence boosting T lymphocyte activation and proliferation. The SNP rs2476601 presumably acts on interaction between PTPN22 and Peptidyl Arginine Deiminase (PAD), which suggests its involvement in protein citrullination, thereby enhancing anti-CCP antibody levels.

However, the missense SNP rs33996649 implements a conformational change in the active site of the catalytic domain of LYP and decreases both its phosphatase activity and influence over T lymphocyte activation and clonal expansion. The studies in the European population implied the protective properties of SNP rs33996649 [27].

The case-control study from Frontiers in Medicine (2025), which included 229 patients with RA and 239 patients in the control group, indicates that the PTPN22 SNPs rs2476601 and rs2488457 are strongly associated with vulnerability to RA in the Indian population. The SNP rs2476601 C/T (OR = 3.6136; CI = 2.2789 to 5.7300; *p* = 0.0001) and rs2488457 C/C genotype (OR = 1.5179; CI = 1.0453 to 2.2042; *p* = 0.0283) are linked with RA. Moreover, the SNP rs2476601 C/T genotype increased the level of anti-cyclic citrullinated peptide (anti-CCP) antibody (*p* = 0.01) [27].

The decreased regulation of PTPN22 expression in RA patients was also observed in Scientific Reports (2017). Eighty-nine patients with RA carrying the risk alleles (PTPN22 rs2488457, rs2476601) (*p* = 0.004) presented the dysregulation compared to 43 healthy controls (*p* = 0.007) [31].

The LYP expression is increased in neutrophils and contributes to reactive oxygen species (ROS) production through NADPH oxidase priming and thus can act proinflammatory [32]. It also leads to susceptibility to citrullination [33].

#### 4.2.2. STAT4

The STAT family plays a considerable role in cytokine-induced cellular responses. One of the members most significant to the autoimmune response is 6-domain STAT4 (Signal Transducer and Activator of Transcription 4), which functions in the Janus kinase (JAK)-STAT pathway, which promotes nuclear translocation (N-terminal domain), binds to regulatory factors (coiled-coil domain), and activates the transcription process (C-terminal domain) [34]. Furthermore, STAT4 contributes to T-helper type 1 and T-helper type 17 lymphocyte differentiation, production of interferon-γ, and the monocyte activation process [35].

The case-control study from Genes (2024) performed on 476 samples (including 240 case group and 236 control group) indicates that the rs7574865 variant of the STAT4 gene with the GT genotype (OR = 2.424, 95% CI 1.268–4.635) and TT genotype (OR = 3.967, 95% CI 1.597–9.854) may be correlated with susceptibility to RA [36].

Another study relying on a genome-wide association study (GWAS) (31 313 RA cases (68% seropositive) and approximately 1 million controls) shows that a missense variant (rs140675301-A) elevates the susceptibility to seropositive RA (*p* = 2.1 × 10^−9^) [18].

#### 4.2.3. CTLA4

Cluster of differentiation 152 (CD152), also referred to as cytotoxic T-lymphocyte-associated protein 4 (CTLA4), is a protein which can preserve organism homeostasis by regulating exaggerated immunological response through attachments between CD80, CD86, antigen-presenting cells (APC), and itself. Its gene is located on chromosome 2q33. Its SNPs are associated with numerous of conditions such as T1D, tuberculosis, and hepatitis types B and C [37].

There are some noteworthy polymorphisms in RA predisposition. Depending on the recessive, heterozygous, and dominant models, where rs733618 and rs11571319 (AA genotype) increase the risk, rs11571315, rs11571316, and rs16840252 of CTLA4 may act protectively [38].

In addition, the CLTA4 protein may play a role as immune checkpoint regulator [39].

#### 4.2.4. TNFAIP3

Tumour necrosis factor α-induced protein 3 (TNFAIP3) gene encodes the A20 enzyme responsible for (de)ubiqitination in the nuclear factor-κB (NF-κB) signalling pathway [40].

In mouse models, the deletion of A20/TNFAIP3 gene induced an inflammatory disease resembling RA. Consequently, macrophages boost caspase-1 activation mediated by NLRP3 and increase IL-1β level, accompanied by pyroptosis [41].

The research comparing RA and osteoarthritis (OA) knee joint synovium has helped identify the location of TNFAIP3, in mostly nuclear (OA patients) and predominately cytoplasmic (RA patients). The SNPs rs6920220, rs6933404, rs6927172 have shown signs of repressor activity towards TNFAIP3 transcription [42].

Different SNPs present distinct effects on the susceptibility to RA. Rs6920220 predisposes a person to seropositive RA, while rs10499194 promotes seronegative RA in relation to anti-CCP antibodies. Rs10499194 seems to confer a protective effect (OR = 0.52) [43].

#### 4.2.5. TRAF1/C5

Due to their shared genomic location at chromosome 9q33–34, the research over complement component 5 (C5) and TNF receptor-associated factor 1 (TRAF1) has been conducted collectively. Polymorphism in this region enhances the predisposition to and severity of RA, especially in anti–CCP-positive patients. The SNPs showing a strong association are rs376147 and rs2900180 [44,45].

Further investigation of TRAF1/C5 is crucial due to the role of complement polymorphisms in RA pathogenesis. SNPs such as rs17611, which codes for V802I polymorphism in C5, sustain the inflammatory process by interfering with the functionality of elastase. The C5V802 variant is prone to cleavage by human neutrophil elastase (HNE) [46].

However, some mutations in TRAF1 may attenuate the inflammatory process, e.g., V203A in humans and V196A in mice. The reduced production of proinflammatory mediators in macrophages is the effect of impaired associations with cellular inhibitor of apoptosis 2 (cIAP2) [47].

A gene localised on chromosome 1q21–23 [48] presenting homology to Fcγ receptors, namely Fc receptor–like protein 3 (FCRL3), is expressed on B, T, and NK cells, notably on the CD4+ FOXP3+ regulatory T cells (Tregs). It fulfils the function of the type I transmembrane protein. It contains noteworthy elements such as Ig-like domains and immunoreceptor tyrosine-based activation motifs (ITAMs) as well as immunoreceptor tyrosine-based inhibitory motifs (ITIMs). FCRL3 acts as an immunomodulator with predominantly inhibitory signalling properties.

The SNP rs7528684 (−169T/C) is [clinically] significant due to its correlation with the overexpression of FCRL3, which inactivates protein tyrosine kinases including LCK and ZAP70, leading to impaired TCR signalling. The result is reduced expression of the forkhead box protein 3 (FOXP3) gene and interleukin-35 (IL-35), diminishing the suppressive activities of Tregs.

According to the research, the C/C genotype of the FCRL3 SNP rs7528684 is significantly linked to RA in the Indian population (*p* = 0.0005), with elevated C allele frequency presentation (58.6%) in RA group. Moreover, the C/C genotype of rs7528684 may be correlated with increased serum IgG-RF level, an autoantibody and an inflammation biomarker [49,50].

### 4.3. Genes Related to Cytokines and Inflammation

#### 4.3.1. IL6R

Interleukin-6 (IL-6) is a cytokine characterised by pleiotropic activity. It is critical for T-cell activation and differentiation. Nevertheless, IL-6 enhances proinflammatory responses, which can lead to autoaggressive diseases.

Depending on the cellular localisation of the IL-6 binding factor, this connection may result in the assumption of a different signalling pathway. Classical signalling relies on the association between IL-6 and a membrane-bound IL-6 receptor (IL-6R), whereas “trans-signalling” rests upon binding IL-6 to a floating soluble form of IL-6R (sIL-6R). Interaction between IL6, IL-6R, and co-receptor IL-6ST (encoding for GP130 protein) [51] induces the development of phosphorylated factors STAT3 and STAT1.

A functional SNP rs222814 (IL6R Asp358Ala) impairs the classical signalling pathway, which has a protective effect on coronary heart disease (CHD), abdominal aortic aneurysm (AAA), and RA, but predisposes a person to asthma [52].

Both seropositive and seronegative RA are linked to reduced ANKRD55 and IL-6ST expression due to the intronic variant rs7731626-A in ANKRD55 [18].

Rs2228145 (IL-6R) and rs1800795 (IL-6) are correlated with anti-CCP positive patients [18].

#### 4.3.2. IL2RA

IL2RA (located on 10p15.1) encodes the α subunit of the IL-2 receptor (CD25), which increases the affinity between IL-2 and the receptor (IL-2R) complex. Interestingly, the IL-2 pathway has a dual role in the matter of autoimmune disease development. It enhances the inflammation by activating effector T cells, so IL-2 deficiency leads to delayed onset of autoimmune response. However, a lack of IL-2 impairs Treg development which contributes to impaired immune tolerance, thus facilitating autoimmunity [53,54].

The studies analysing DMARD—free sustained remission implied that a IL2RA-rs2104286 minor allele increased the probability of remission (HR = 1.52, 95% CI = 1.16–1.99, *p* = 2.44 × 10^−3^) due to lower sIL2Rα-levels. Additionally, IL2RA-rs2104286 is the only genetic factor except HLA-SE implicated in RA development and persistence [25].

The novel retrospective study (2026) draws attention to the potential of distinguishing treatment-naive RA (TN-RA) from undifferentiated arthritis (UA) at the disease onset. They differ in diminished IL-2 responsiveness and global inflammatory state (TN-RA) versus Th17/Treg imbalance (UA). The main purpose of this research is to potentially simplify the decision-making process and treatment approach at an early stage of the disease [55].

#### 4.3.3. CCR6

A receptor for the C–C motif chemokine ligand-20 (CCL20) is the ligand of C–C motif chemokine receptor-6 (CCR6), and they show codependence through the CCR6-CCL20 axis. CCR6’s key roles are to recruit Th17, suppress Treg, and impact the chemotactic activity of B cells, T cells, and dendritic cells [56].

However, the IL-1β, tumour necrosis factor α (TNF-α) and, expressed by Th17, IL-17 amplify the CCL20 production, which leads to a positive feedback loop and sustains the inflammatory process [57].

CCR6-CCL20 signalling may be involved in RA development.

IL2RA and CCR6 have been described as genes which increase susceptibility to ACPA-positive RA [43].

The data have demonstrated that in comparison to OA patients, the synovial fluid of RA patients show the presence of higher amounts of IL-17 and CCL20. However, further research is necessary to determine whether therapies targeting Th17 migration would be beneficial in RA treatment [57].

This ligand-receptor pair draws attention towards the search for new treatment strategies aimed at other autoimmune diseases, such as psoriasis (PsO) and psoriatic arthritis (PsA) [58].

In mouse models, a correlation has been discovered between fibroblast-like synoviocyte (FLS) and the classical myokine myostatin/growth differentiation factor 8 (GDF-8), which can sustain the inflammatory process, due to its impact on Th17. Thus, targeting myostatin could prove to be another therapeutic aim to weaken inflammation [59].

Up-to-date data (2026) are oriented to innovative strategies called “immunological surgery” depending on Fc-engineered anti-human CCR6 monoclonal antibody (αhCCR6 DLE-mut mAb) directed towards eliminating CCR6+ cells.

In bleomycin-induced scleroderma, imiquimod-induced psoriasis, and collagen-induced arthritis (CIA) models there has been observed an improvement in consecutive skin inflammation and fibrosis; increased skin thickness and dermal leukocyte infiltration; and the manifestation of joint inflammation. Nevertheless, despite promising therapeutic potential, other CCR6+ populations should be further examined to comprehensively characterise the immune profile [60].

### 4.4. Newly Discovered Genetic Loci

GWAS contribute to advances in research into susceptibility loci and due to the analysis of pathology mechanisms, directions for further therapies.

Regarding the IL6R locus, the interesting variants linked to RA are rs12126142 and rs4341355. Despite their weak correlation, they differ in mechanism of action (MoA). Both of them have an impact on IL6R function; rs4341355 acts on IL6R splicing and rs12126142 affects total IL6R expression [6].

The missense variant rs2269495 (A313V in TNIP2) leads to impaired function of the TNIP2 protein. It interferes with TNF-induced NF-κB inhibition, which may contribute to RA pathology [6].

A GWAS study from 2022 not only links seropositive RA susceptibility with the rs140675301-A STAT4 variant, but also with a stop-mutation (rs76428106-C) in FLT3 (OR = 1.35, *p* = 6.6 × 10^−11^). However, different variants may be connected with a reduced risk of seropositive RA, for example TYK2 rs34536443-C, rs12720356-C, and rs35018800-A (Ors = 0.63–0.87, *p* = 10^−9^–10^−27^) [18].

Some studies focus on identifying the core genes involved in rheumatoid arthritis. The Arthritis & Rheumatology study from 2025 suggests the roles of CD5, CTLA4, TIGIT, LAIR1, PDCD1, and TNFRSF14 as immune checkpoints. Other genes (LGALS9, IL2RA, TNFRSF9, CD274, and CD27) analysed on the basis of mouse models can participate in the inflammatory process [39].

Considering TNFAIP3 (A20), the rs6920220 located on 6q23 was identified as the genetic variant associated with RA, followed by rs6933404 and rs6927172. This one disturbs negative regulation of NF-κB, thus impairing cytokine expression and proinflammatory activity [39]. Additionally, the rs6920220 SNP was observed to increase the susceptibility to systemic lupus erythematosus (SLE) (*p* = 0.03, OR = 1.53) and primary Sjögren’s syndrome (pSS) (*p* = 0.016, OR = 1.69) [61].

The SNPs linked to TRAF1 not only confer an increased risk of RA, but juvenile idiopathic arthritis and mortality from sepsis as well. The recent study from Journal of Autoimmunity (2025) analysing V203A sheds new light on therapeutic strategies relying on TRAF1. The profound analysis suggests that medicines targeting Traf1/cIAP2 association may become a new effective strategy, especially for resistant patients [47].

Transcription factors (TF) are proteins which attach to the specific DNA sequences and act as regulators. TF are responsible for inducing and silencing gene expression.

## 5. Biological and Clinical Significance of Polymorphisms

### 5.1. Pharmacogenomics and Personalised Medicine

Gene polymorphisms may alter virtually every aspect of pharmacokinetics and pharmacodynamics. The assessment of polymorphisms affecting the action of DMARDs may be the next crucial step in the development of more personalised therapies for RA.

Currently, methotrexate is the first-line treatment in newly diagnosed RA, and several gene polymorphisms were shown to alter the response to this DMARD [62]. The mechanism of action of methotrexate in RA is complex and the effect on adenosine signalling is one of its most important elements. Methotrexate inhibits an enzyme called AICAR transformylase, which causes intracellular accumulation of AICAR, thereby leading to the inhibition of another enzyme adenosine deaminase and increase in intracelullar adenosine. Adenosine is transported out of the cells and binds its receptors on the cell surface (including ADORA2A and ADORA3), which facilitates the anti-inflammatory effects of methotrexate [63]. It was demonstrated that polymorphisms in adenosine receptors ADORA2A and ADORA3 affect the efficacy of methotrexate in RA. The carriage of the T allele of the ADORA2A rs17004921 and the C allele of ADORA3 rs2298191 was associated with a greater decrease in disease activity at a 6 month follow-up [63,64]. Receptor signalling via both ADORA2A and ADORA3 is known to have anti-inflammatory effects in macrophages. ADORA2A activation was demonstrated to suppress the production of neutrophil migration factors via macrophages engulfing apoptotic cells. The stimulation of ADORA3 was shown to hamper production of TNFα, inducible nitric oxide synthase, and interleukin-1beta [65,66]. In a study on Indian patients, rs1127354 polymorphism of the Inosine triphosphate pyrophosphatase (ITPA) gene was also associated with the occurrence of a response to methotrexate [67]. ITPA is an enzyme hydrolysing ITP to IMP and diphosphate, and thereby it may affect adenosine metabolism [68]. The adenosine signalling, on the other hand, is implicated in the mechanism of action of methotrexate in RA, which may explain the effects of rs1127354 on RA therapy.

According to EULAR recommendations, the biological DMARDs (preferably combined with conventional synthetic DMARD) are the second-line treatment in RA. This group of drugs includes sarilumab, TNFα inhibitors (infliximab, adalimumab, certolizumab, golimumab, etanercept), rituximab, abatacept, and tocilizumab [62]. Similar to methotrexate, the clinical response to TNFα inhibitors may be predicted via genotyping SNPs. For example, in a study by Sánchez-Maldonado et al., LINC02549 rs7767069 was associated with a worse clinical response to TNFα inhibitors (OR (95% CI) = 0.85 (0.76–0.96), *p* = 0.008) [69]. Another SNP FTO rs7195994 was found to be an independent predictor of TNFα inhibitors inefficacy (OR = 0.44, 95% CI = 0.22–0.87; *p* = 0.019). Interestingly, when patients were stratified by BMI, the association of FTO rs7195994 with TNFi response was significant only in patients with BMIs below 27. This finding is in accordance with the known role of the FTO gene in adipose tissue metabolism, however the exact mechanisms were not investigated and further research on this matter is warranted [70]. Latini and colleagues showed that another SNP, VDR rs11568820 (C > T) is associated with response to TNFi. The VDR gene encodes the vitamin D receptor, and the role of this vitamin in RA outcomes is well established. However, the authors did not investigate the effect of VDR rs11568820 on vitamin D signalling [71,72].

### 5.2. Markers

Current ACR/EULAR RA classification criteria include four categories: joint involvement, serology, acute phase reactants, and duration of symptoms [68]. The circulating serological markers RF and ACPA, despite being highly (90% specificity for RF) or very highly specific (96% for ACPA), have a relatively low sensitivity (68.6% for RF and 57% for ACPA) [73,74]. Additional testing of genetic predispositions, including gene polymorphisms, could aid the diagnosis of RA, especially since in some cases the differential diagnosis may be challenging. Knevel et al. investigated the use of their original genetic probability model G-PROB to differentiate the diseases causing inflammatory arthritis. The G-PROB model is based on 42 HLA region genetic variants and 208 SNPs outside of the HLA region. G-PROB demonstrated a considerable ability to discriminate between diagnostic entities in patients presenting with inflammatory arthritis at the first visit to rheumatologist (AUC = 0.84 (95% CI, 0.81 to 0.86)) [75]. The discriminative ability of this genetic probability model was corroborated in a latter study by Hum et al. using pooled AUC (95% CI) = 0.85 (0.84–0.86) [76].

Another potential clinical application of polymorphism genotyping is the identification of individuals at risk of developing rheumatoid arthritis, which may allow for early diagnosis and introduction of treatment. The information on RA-risk polymorphisms in a patient may be used to calculate the patient’s polygenic risk scores, estimating the individual’s genetic liability to a disease [77]. Lim and collaborators used machine learning (ML) to create a polygenic risk score for RA based on 9 SNPs. This ML-optimised polygenic risk score demonstrated an excellent predictive value with an AUC, sensitivity, and specificity above 0.9 for three previously unseen datasets [78]. Xu and Wu evaluated the predictive accuracy of four ML models for RA risk in postmenopausal women. The model utilising conventional risk factors and SNPs with kernel principal component analysis and using the XGBoost method had the highest predictive value, with an AUC of 0.9 [79] (Figure 3). Beyond SNP-based risk prediction, machine learning has also been applied to whole-blood transcriptomic data to identify biomarkers of treatment response. An AI-optimised analysis of pre-treatment whole-blood transcriptomes identified MZB1 expression as a novel predictor of both antidrug antibody formation and clinical response to adalimumab, illustrating the extension of ML-based approaches from genetic risk stratification to prediction of therapeutic outcome [80].

### 5.3. Polymorphisms and Pathophysiology of RA

Numerous gene polymorphisms have been shown to be directly involved in the pathogenesis of RA. An example of such RA-risk polymorphism is rs11701653, located in the CD28/CTLA4/ICOS locus. Mechanistically, the carriage of the rs11701653 A allele decreases the binding of SMCHD1, which prevents the suppression of the inducible T-cell costimulator (ICOS) in memory T cells. SMCHD1 is an inhibitory chromatin regulator which binds DNA directly through its C-terminal SMC hinge domain [81]. The exact mechanism of action of SMCHD1 in this case was not investigated. ICOS stimulation of memory CD4+ T cells promotes TGF-beta-induced differentiation into CXCR5-PD-1highTph cells, which were shown to be involved in RA pathogenesis through the production of chemokine (C-X-C motif) ligand 13 (CXCL13), a molecule attracting B cells and Interleukin-21, a cytokine promoting the differentiation of plasmablasts [82]. Chemokine receptor 6 (CCR6) gene dinucleotide polymorphism is another known RA susceptibility polymorphism [83]. The CCR6 double nucleotide polymorphism TG allele is preferably bound by poly (ADP-ribose) polymerase 1 (PARP1), which facilitates CCR6 gene expression through its enzymatic activity [84]. CCR6, through binding of chemokine (C-C motif) ligand 20 (CCL20), promotes migration of mononuclear cells (including Th17) to the inflamed joints [85].

Fibroblast-like synoviocytes (FLSs) play a crucial role in rheumatoid arthritis by producing proangiogenic factors, proinflammatory cytokines, and metalloproteases. FLSs in RA are characterised by reduced apoptosis, increased migration, and invasiveness [86]. These characteristics of FLSs are possibly promoted by the C allele of rs26232, which is known to be associated with susceptibility to RA and disease severity. Sheridan investigated the mechanism behind this effect in an in vitro model, demonstrating that it may be explained by a decrease in the expression of an enzyme called peptidylglycine alpha-amidating monooxygenase (PAM) in RA FLSs. Lower expression of PAM results in lower amidation of the FLS-secreted signalling peptides adrenomedullin (ADM) and pro-ADM N-terminal peptide (PAMP), which leads to increased invasiveness and proliferation of FLSs. Moreover, inhibition of the amidation of these FLS-secreted molecules skews macrophages toward a proinflammatory MerTK phenotype. The increased invasiveness of FLSs and promotion of MerTK-phenotype macrophages facilitates joint destruction in RA [87]. On the other hand, the T allele of rs1876518 in the intronic region of the SPRED2 gene was shown to reduce the proinflammatory characteristics FLSs. Transcription factor CCAAT/enhancer-binding protein beta (CEBPB) preferentially binds to this allele, allowing for the expression of the Sprouty-related, EVH1 domain-containing protein 2 (SPRED2). The exact mechanism of action of CEPBP in this case is uncertain. However, it is known to be able to recruit co-activators and simple transcriptional factors [88]. SPRED2 creates a positive feedback loop with another molecule, actin-related protein 2 (ACTR2), and increased expression of these proteins reduces the migration, invasiveness, and proliferation of FLSs. Interestingly, ACTR2 is a key element of the ARP2/3 complex, which is known to initiate the formation of daughter F-actin filaments. However, it is unknown whether this plays a role in the mechanism of action of ACTR2 in RA [89]. SPRED2 may also act through its effect on actin cytoskeleton, however further research on the exact mechanism of action of this protein in RA is needed [90].

Table 1 summarises the key gene polymorphisms discussed above, together with their principal immunological mechanism and clinical or pharmacogenomic relevance, to help organise this large body of evidence. For each locus, the table also indicates the type of evidence on which the association rests, the population or ancestry in which it was reported, and the RA phenotype with which it is associated.

## 6. Discussion, Limitations, and Future Directions of GWAS Studies in RA

Despite significant progress in identifying genetic risk factors for RA, GWAS do not fully explain the complex genetic architecture of the disease. One of the main challenges remains the phenomenon of ‘missing heritability’—that is, the difference between the heritability of RA estimated from familial studies and the variance explained by identified loci. This phenomenon stems from, among other factors, the contribution of numerous variants with a small effect, rare genetic variants, structural genomic variations, and gene–gene and gene–environment interactions. Classical GWAS also have a limited ability to identify rare and non-coding variants that may play a significant role in regulating gene expression.

Another limitation is population heterogeneity. A significant portion of GWAS have been conducted in populations of European descent, while the allele frequencies, LD structure, and effect sizes of individual variants may differ between populations. The limited representation of different ethnic groups may hinder the replication of results and reduce the accuracy of polygenic risk models in underrepresented populations.

Interpreting GWAS results also remains a significant challenge. Detecting a statistically significant association does not directly indicate the identification of a causal variant. A variant may be in LD with the actual functional variant, and many loci encompass multiple genes and regulatory elements. Variants located in non-coding regions are particularly important, as they can influence gene expression by interacting with promoters, enhancers, or transcription factors. Therefore, GWAS results require confirmation by functional studies. Their interpretation should also consider epigenetic mechanisms, such as DNA methylation, histone modifications, and changes in chromatin accessibility, which may link genetic predisposition to the influence of environmental factors.

Future studies should therefore go beyond traditional GWAS and utilise next-generation sequencing, including whole-genome and exome sequencing, which enables the identification of rare variants and structural changes. Integrating genomic data with transcriptomic, epigenomic, proteomic, and metabolomic information will be particularly important. A multi-omics approach can allow for better definition of the relationship between genetic variants, gene expression, and specific RA phenotypes. Furthermore, the use of artificial intelligence and ML methods can facilitate the analysis of multidimensional datasets and the identification of complex relationships between genetic, molecular, and clinical factors.

In the long term, integrating genetic and multi-omics data could contribute to the development of precision medicine in RA. Identifying genetic and molecular profiles associated with disease risk, course, and treatment response could enable better patient stratification and more personalised therapy selection. However, achieving this goal will require large, population-diverse cohorts, functional studies, and independent validation of potential biomarkers.

In summary, GWAS have played a crucial role in identifying loci associated with RA susceptibility and have contributed to a better understanding of the molecular mechanisms of the disease. At the same time, their limitations point to the need to integrate genetic data with other levels of biological regulation. Combining genomics, epigenomics, transcriptomics, proteomics, and modern data analysis methods may, in the future, enable more effective use of genetic discoveries in the diagnosis, prognosis, and personalisation of RA therapy.

## Figures and Tables

**Figure 1:**
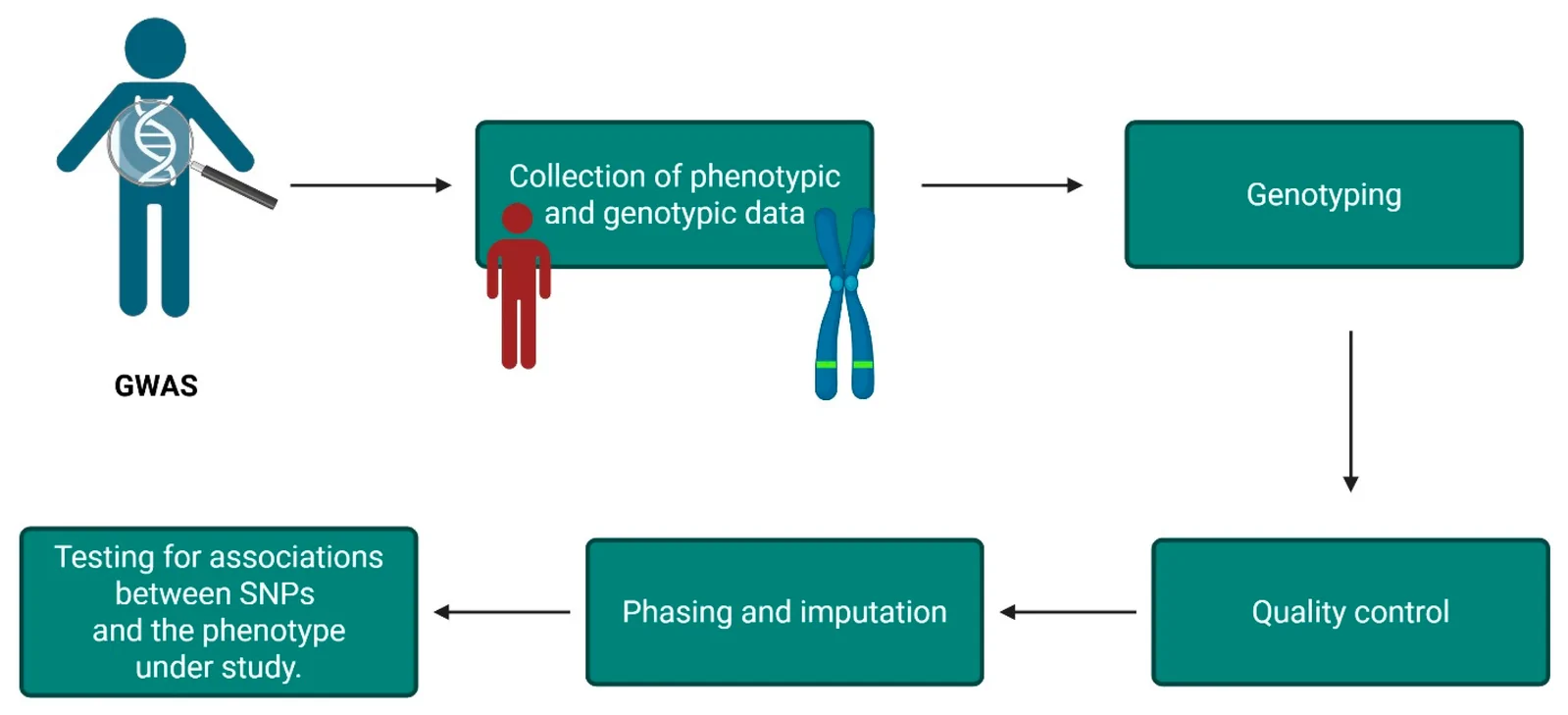
GWAS stages. Created in BioRender. Plewa, P. (2026) https://BioRender.com/5smukdw, accessed on 21 September 2026.

**Figure 2:**
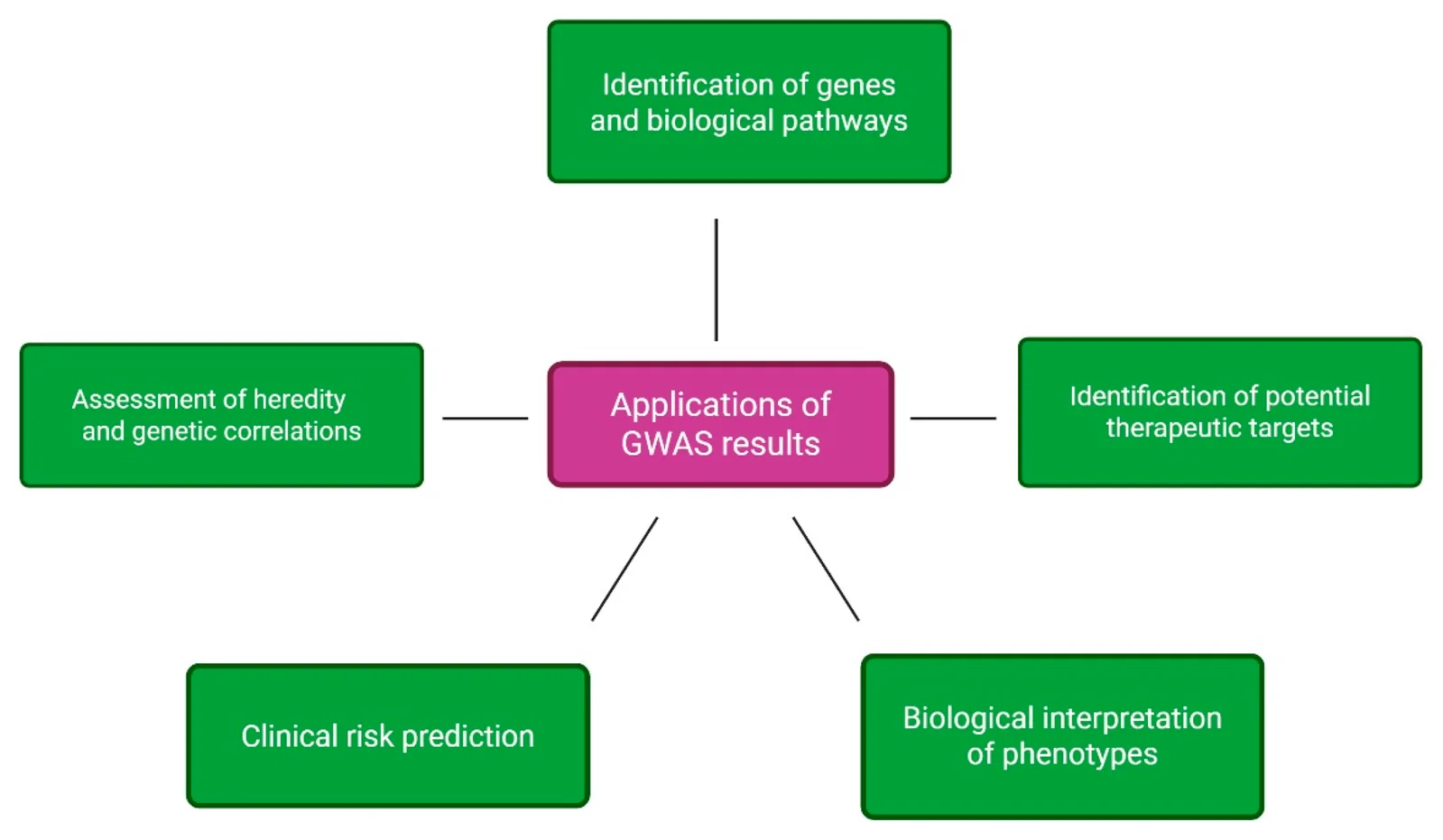
Main applications of GWAS results. Created in BioRender. Plewa, P. (2026) https://BioRender.com/kte16qa, accessed on 21 September 2026.

**Figure 3:**
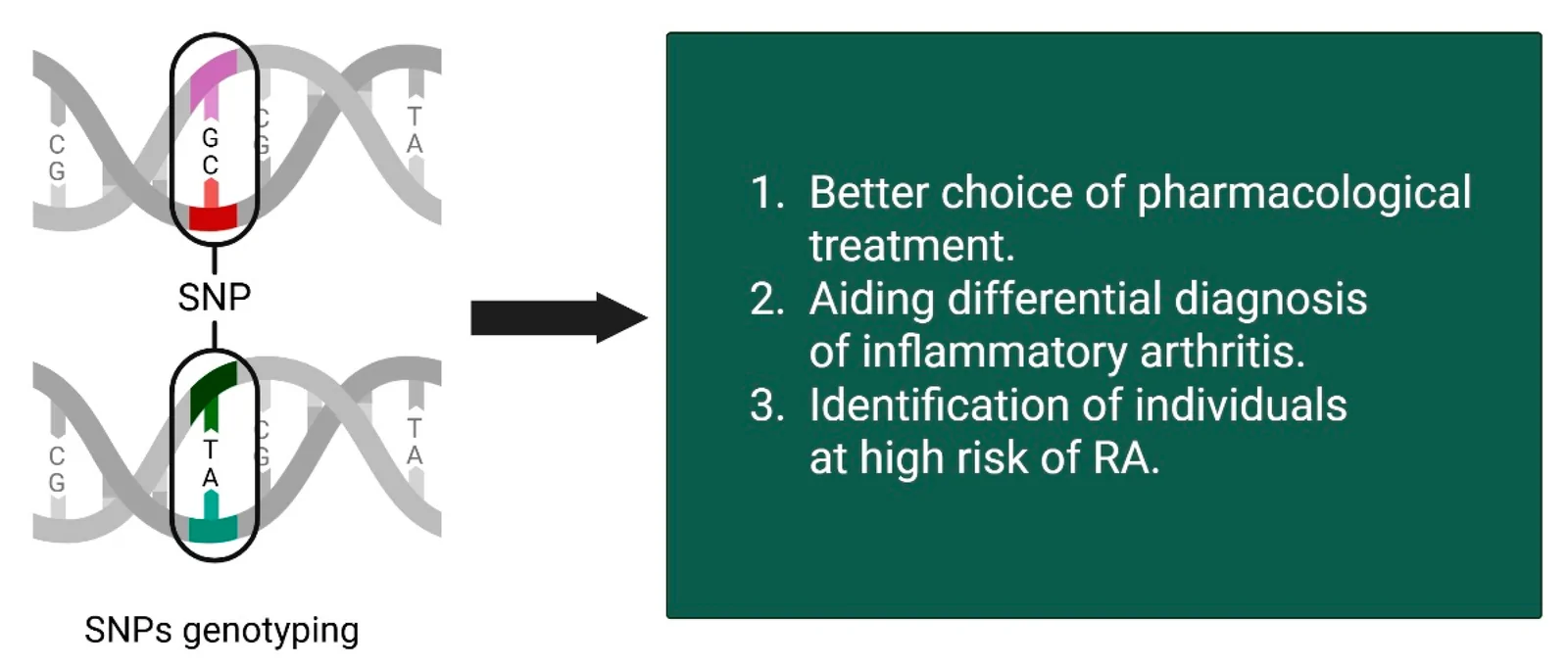
Clinical applications of SNPs genotyping in RA. Created in BioRender. Szulc, J. (2026) https://BioRender.com/glos8ca, accessed on 21 September 2026.

**Table 1:** Summary of key rheumatoid-arthritis-associated gene polymorphisms discussed in this review. Type of evidence, study population/ancestry and the associated RA phenotype are given for each locus; “GWAS” denotes genome-wide association studies and meta-analyses thereof, as distinct from candidate-gene, functional/preclinical and pharmacogenomic studies.

Gene/Locus	Primary SNP(s)	Risk/Protective Allele	Type of Evidence	Population/Ancestry	Associated RA Phenotype	Primary Immunological Mechanism	Clinical/Pharmacogenomic Implication	References
HLA-DRB1	Shared-epitope haplotypes (*04:01/*04:04/*04:05/*04:08, *10:01, *01:01)	Risk; *13 and *11 group protective	GWAS and HLA fine-mapping; candidate-allele association studies	European (UK); Chilean; multi-ethnic Asian (Malay, Chinese, Indian)	ACPA-positive (seropositive) RA; radiographic severity; mortality; DMARD-free remission	Shared-epitope-mediated MHC-II presentation of citrullinated peptides to CD4+ T cells	Marker of seropositive (ACPA+) RA, disease severity, and mortality risk	[21,22,23,24]
PTPN22	rs2476601 (R620W)	Risk (T allele)	Candidate-gene case-control studies; functional (gene-editing, cellular) studies	European; Indian	Susceptibility; anti-CCP-positive RA	Dysregulated LYP-mediated negative regulation of TCR signalling	Susceptibility and anti-CCP positivity marker	[27,28,29,30,31,32,33]
STAT4	rs7574865; rs140675301	Risk	Candidate-gene case-control study (rs7574865); large-scale GWAS (rs140675301)	Mexican; Northern European	Susceptibility; seropositive RA	JAK–STAT pathway; Th1/Th17 differentiation	Susceptibility marker, seropositive RA	[18,34,35,36]
CTLA4	rs733618, rs11571319 (risk); rs11571315, rs11571316, rs16840252 (protective)	Risk/Protective	Candidate-gene case-control study	East Asian (Taiwanese)	Susceptibility	Altered CD80/CD86 immune checkpoint co-stimulation	Susceptibility marker	[37,38,39]
TNFAIP3 (A20)	rs6920220 (risk, seropositive); rs10499194 (protective, seronegative)	Risk/Protective	Candidate-gene association studies; functional and murine studies	European (Spanish, UK, Italian)	Seropositive RA (rs6920220); seronegative RA (rs10499194)	Impaired NF-κB negative regulation via A20 deubiquitination	Susceptibility marker; shared with SLE and pSS	[40,41,42,43]
TRAF1/C5	rs3761847, rs2900180, rs17611 (risk); TRAF1 V203A (protective)	Risk/Protective	GWAS and candidate-gene studies; functional and preclinical studies	European (Dutch, Swedish, North American of European ancestry)	ACPA-positive RA; disease severity	Complement (C5–elastase) and TRAF1–cIAP2 signalling	Severity marker; TRAF1–cIAP2 axis proposed as therapeutic target	[44,45,46,47,48,49,50]
IL6R	rs2228145 (Asp358Ala)	Protective for RA; risk for asthma	Large-scale genetic association study with functional characterisation	European	Susceptibility (protective); anti-CCP-positive RA	Impairs classical IL-6R signalling	Pleiotropic cross-disease marker	[18,51,52]
IL6R (regulatory)	rs12126142, rs4341355	Risk	GWAS with expression/splicing (eQTL) annotation	European/multi-ancestry	Susceptibility	Altered IL6R splicing/expression	—	[18,51,52]
ANKRD55/IL6ST	rs7731626	Risk	Large-scale GWAS and multi-omics analysis	Northern European (Iceland, Denmark, Sweden, Norway)	Seropositive and seronegative RA	Reduced ANKRD55/IL-6ST expression	Susceptibility marker	[18,19]
IL2RA	rs2104286	Protective	Prognostic cohort (candidate-gene) study; functional studies	European	DMARD-free sustained remission; disease persistence	Lower soluble IL-2Rα, supports Treg-mediated tolerance	Predicts DMARD-free sustained remission	[25,53,54,55]
CCR6	CCR6–CCL20 dinucleotide (TG allele)	Risk	GWAS; functional and preclinical (murine) studies	East Asian (Japanese); European	ACPA-positive RA susceptibility	PARP1-enhanced CCR6 expression, Th17 recruitment	Susceptibility marker, ACPA + RA	[43,56,57,58,59,60]
TNIP2	rs2269495 (A313V)	Risk	Large-scale GWAS (missense variant)	Northern European	Susceptibility	Impairs TNF-induced NF-κB inhibition	—	[6]
CD28/CTLA4/ICOS locus	rs11701653	Risk (A allele)	Functional fine-mapping of a GWAS-identified non-coding variant	European	Susceptibility	Derepresses ICOS on memory T cells, driving Tph-cell CXCL13/IL-21 production	—	[81]
PAM locus	rs26232	Risk (C allele)	GWAS-identified variant with in vitro functional validation	European	Susceptibility; disease severity (joint damage)	Reduced PAM amidase activity increases synoviocyte invasiveness	Severity marker	[87]
SPRED2	rs1876518	Protective (T allele)	Functional/enhancer study of a GWAS-identified variant	Not specified in the source study	Susceptibility (protective)	CEBPB-driven SPRED2 expression limits synoviocyte migration/invasiveness	—	[90]
ITPA	rs1127354	Pharmacogenomic	Pharmacogenomic candidate-gene study	Indian	Methotrexate response	Altered adenosine metabolism	Predicts methotrexate response	[67,68]
ADORA2A/ADORA3	rs17004921 (ADORA2A); rs2298191 (ADORA3)	Pharmacogenomic	Pharmacogenomic candidate-gene study; preclinical mechanistic studies	European	Methotrexate response (disease activity at 6 months)	Anti-inflammatory adenosine-receptor signalling	Predicts methotrexate response	[63,64,65,66]
LINC02549	rs7767069	Pharmacogenomic (poor response)	Pharmacogenomic validation of GWAS-identified variants (meta-analysis)	European	TNFα-inhibitor response	Regulatory mechanism not established	Predicts worse TNFα-inhibitor response	[69]
FTO	rs7195994	Pharmacogenomic	Pharmacogenomic cohort study	East Asian (Taiwanese)	TNFα-inhibitor response (BMI < 27)	Adipose tissue metabolism (BMI-dependent)	Predicts TNFα-inhibitor inefficacy in patients with BMI < 27	[70]
VDR	rs11568820	Pharmacogenomic	Pharmacogenomic candidate-gene study	European (Italian)	TNFα-inhibitor response	Vitamin D receptor gene variant (mechanism not established)	Predicts TNF-inhibitor response	[71,72]

## Data Availability

No new data were created or analysed in this study. Data sharing is not applicable to this article.

## Abbreviations

The following abbreviations are used in this manuscript:

ACPA	Anti-citrullinated protein antibodies
anti-CCP	Anti-cyclic citrullinated peptide
AMD	Age-related macular degeneration
AMPA	Anti-modified protein antibodies
APC	Antigen-presenting cell
BMI	Body mass index
CD	Cluster of differentiation
DMARD	Disease-modifying antirheumatic drug
CTLA4	Cytotoxic T-lymphocyte-associated protein 4
FLS	Fibroblast-like synoviocyte
GWAS	Genome-wide association studies
HLA	Human Leukocyte Antigens
HNE	Human neutrophil elastase
IL	Interleukin
ITPA	Inosine triphosphate pyrophosphatase
JAK	Janus kinase
LD	Linkage disequilibrium
LYP	Lymphoid-Specific Tyrosine Phosphatase
MHC	Major histocompatibility complex
PTPN22	Protein Tyrosine Phosphatase Non-Receptor Type 22
RA	Rheumatoid arthritis
RF	Rheumatoid factors
SNP	Single-nucleotide polymorphism
STAT	Signal Transducer and Activator of Transcription
TNFAIP3	Tumour necrosis factor α-induced protein 3
TRAF1	TNF receptor-associated factor 1
